# What is a preferred angiotensin II receptor blocker-based combination therapy for blood pressure control in hypertensive patients with diabetic and non-diabetic renal impairment?

**DOI:** 10.1186/1475-2840-11-32

**Published:** 2012-04-10

**Authors:** Samir G Mallat

**Affiliations:** 1 Division of Nephrology and Hypertension, Department of Internal Medicine, Faculty of Medicine, American University of Beirut, Beirut, Lebanon

**Keywords:** Amlodipine, Angiotensin receptor II blocker, Diabetes mellitus, Hydrochlorothiazide, Hypertension, Renal impairment, Single-pill combination, Telmisartan

## Abstract

Hypertension has a major associated risk for organ damage and mortality, which is further heightened in patients with prior cardiovascular (CV) events, comorbid diabetes mellitus, microalbuminuria and renal impairment. Given that most patients with hypertension require at least two antihypertensives to achieve blood pressure (BP) goals, identifying the most appropriate combination regimen based on individual risk factors and comorbidities is important for risk management. Single-pill combinations (SPCs) containing two or more antihypertensive agents with complementary mechanisms of action offer potential advantages over free-drug combinations, including simplification of treatment regimens, convenience and reduced costs. The improved adherence and convenience resulting from SPC use is recognised in updated hypertension guidelines. Despite a wide choice of SPCs for hypertension treatment, clinical evidence from direct head-to-head comparisons to guide selection for individual patients is lacking. However, in patients with evidence of renal disease or at greater risk of developing renal disease, such as those with diabetes mellitus, microalbuminura and high-normal BP or overt hypertension, guidelines recommend renin-angiotensin system (RAS) blocker-based combination therapy due to superior renoprotective effects compared with other antihypertensive classes. Furthermore, RAS inhibitors attenuate the oedema and renal hyperfiltration associated with calcium channel blocker (CCB) monotherapy, making them a good choice for combination therapy. The occurrence of angiotensin-converting enzyme (ACE) inhibitor-induced cough supports the use of angiotensin II receptor blockers (ARBs) for RAS blockade rather than ACE inhibitors. In this regard, ARB-based SPCs are available in combination with the diuretic, hydrochlorothiazide (HCTZ) or the calcium CCB, amlodipine. Telmisartan, a long-acting ARB with preferential pharmacodynamic profile compared with several other ARBs, and the only ARB with an indication for the prevention of CV disease progression, is available in two SPC formulations, telmisartan/HCTZ and telmisartan/amlodipine. Clinical studies suggest that in CV high-risk patients and those with evidence of renal disease, the use of an ARB/CCB combination may be preferred to ARB/HCTZ combinations due to superior renoprotective and CV benefits and reduced metabolic side effects in patients with concomitant metabolic disorders. However, selection of the most appropriate antihypertensive combination should be dependent on careful review of the individual patient and appropriate consideration of drug pharmacology.

## Introduction

Hypertension is a highly prevalent disease with a major associated risk for cardiovascular (CV) morbidity and mortality [1-3]. The majority of patients with hypertension require more than one antihypertensive agent to achieve and maintain guideline-recommended blood pressure (BP) goals [4-8]. Identifying the most appropriate combination therapy for each patient based on individual risk factors and comorbidities is important for risk management. Increasingly, single-pill combinations (SPCs) containing two or more antihypertensive agents with complementary mechanisms of action are available. These offer potential advantages, including simplification of treatment regimens, more convenient drug administration and reduced healthcare costs [5,9,10]. Evidence from meta-analyses has shown that the use of antihypertensive SPCs compared with corresponding free-drug combinations is associated with significantly greater rates of treatment adherence to medication and potential advantages in terms of BP improvements and adverse effects [11,12]. A large retrospective database study of an angiotensin II receptor blocker (ARB) plus a calcium channel blocker (CCB) in two-drug SPCs has also shown greater levels of adherence compared with the corresponding free-pill ARB/CCB regimens [13]. Treatment adherence is an important issue for a chronic disease such as hypertension, with improvements in adherence expected to result in better long-term clinical outcomes, including reduced CV and renal morbidity/mortality. This review will consider the choice of agents for combination therapy using two-drug SPCs and the rationale for using particular combinations in patients with hypertension and renal impairment.

### Why should early combination therapy be considered?

Worldwide guidelines recommend combination therapy as a first-line treatment option for hypertension likely not to be controlled on monotherapy (e.g. 20/10 mmHg above target BP) because of evidence showing that only a minority of patients will achieve and maintain BP goals on monotherapy [5-8,14]. The recent re-appraisal of the European guidelines also recommended the preferential use of SPCs to improve adherence [7].

There are a number of compelling reasons why early combination therapy should be used in patients with hypertension (Table 1), including lack of efficacy with monotherapy, greater BP control and attenuation of side effects associated with monotherapeutic treatment [15-20]. In addition, hypertensive patients with comorbidities, such as renal disease, might benefit from additional effects of multiple antihypertensive agents, beyond those related to BP lowering [10].

**Table 1:** Rationale for and potential advantages of early SPC antihypertensive therapy [10,15,16,21]

Rationale:	
1.	Monotherapy is not effective at reaching and maintaining BP goal in most patients

2.	Each difference of 20 mmHg usual SBP or 10 mmHg usual DBP is associated with a two-fold increase in vascular death

3.	Using lower doses of each agent reduces the likelihood of adverse events experienced with a single agent used at a higher dose

4.	Patients with comorbidities, such as renal disease, might benefit from the non-BP-lowering benefits of antihypertensive agents with complementary mechanisms of action

**Potential advantages:**	

1.	Simplified treatment regimen, which is particularly relevant in older patients with comorbid diseases requiring complicated polytherapy

2.	Increased adherence and persistence compared with equivalent free-drug combinations

3.	Additive effects on BP control of individual components with different, complementary mechanisms of action

4.	Attenuation of recognised adverse events, such as reduced CCB-induced peripheral oedema and diuretic-induced metabolic changes with RAS blockers

5.	Lower costs through increased BP reductions

Abbreviations: BP = blood pressure; CCB = calcium channel blocker; DBP = diastolic blood pressure; RAS = renin-angiotensin system; SBP = systolic blood pressure; SPC = single-pill combination

### What are the preferred drug classes for combination regimens?

A range of mostly two-drug antihypertensive SPCs is available [10]. Preferred drug classes for combination regimens target the renin-angiotensin system (RAS), such as ARBs and angiotensin-converting enzyme (ACE) inhibitors, CCBs and diuretics, with selection dependent on individual patient factors, including additional CV risk factors and comorbidities [4,7]. For example, in patients with diabetes and high-normal BP or overt hypertension, which together confer a greater risk of renal damage, combination therapy with a RAS blocker is preferred because these agents offer a superior protective effect against initiation and progression of nephropathy [6]. In patients with renal disease, antihypertensive therapy should aim to target a range of markers of renal (and CV) risk, such as serum creatinine, urine albumin:creatinine ratio, microalbuminuria and proteinuria, usually by RAS blockade, with a view to reducing and slowing progression to end-stage renal disease (ESRD) and CV events [6,20]. Microalbuminuria in particular is a marker of global CV risk and is very common in patients with hypertension [22]. Several position statements also recommend combined therapy that includes RAS blockers. The American Society of Hypertension indicated a preference for RAS blockers in combination with either a diuretic or CCB, with SPCs rather than separate agents preferred when convenience outweighs all other considerations [23]. In addition, the International Society on Hypertension in Blacks (IHSB) recommend a RAS blocker-diuretic or CCB combination in patients with BP > 15/10 mmHg above the target goal [24]. IHSB guidance extends to recommending combination with CCB over diuretics where appropriate (in absence of oedema and/or volume-overload states) due to superiority for hard clinical outcomes.

Owing to the CV and renal protective effects of RAS inhibitors, dual RAS blockade is currently under investigation, i.e. ACE inhibitors, ARB combinations and direct renin inhibitor (DRI) combinations. However studies of double RAS blockade in high-risk patients have provided mixed results [25-28] and current evidence therefore, does not support this therapeutic approach [29].

SPCs containing an ARB may be preferred over those containing ACE inhibitors. ARBs have superior tolerability over ACE inhibitors, which inhibit the degradation of bradykinin, leading to adverse effects, such as dry cough and angioedema [30,31]. Several studies have shown that treatment with ARBs is associated with significantly lower rates of cough and angioedema versus ACE inhibitors [32,33]. Furthermore, ARBs (in particular telmisartan) are well tolerated in patients who are intolerant of ACE inhibitors [34]. Due to their superior tolerability, ARBs may be associated with a higher rate of adherence than ACE inhibitors. In a large cohort of patients in Italy, the rate of discontinuation of the initial single antihypertensive drug treatment was lower for ARBs compared with ACE inhibitors (hazard ratio [HR], 0.92; 95% confidence interval [CI], 0.90-0.94) [35].

In addition to increasing the BP-lowering effects of thiazides and CCBs, adding a RAS inhibitor may help to attenuate the unfavourable metabolic side effects of thiazide monotherapy [36] and CCB-induced peripheral oedema [37-40]. CCB-induced peripheral oedema, which is most likely to occur with dihydropyridine calcium antagonists[41], is caused by increased capillary pressure and flow leading to increased permeability and fluid hyperfiltration [42], appears to be abrogated by post-capillary dilation and normalisation of hydrostatic pressure induced by RAS blockers [43]. Although attenuation of CCB-induced oedema may not as great as that seen with ACE inhibitors [44], ARBs may still be the preferred choice of RAS inhibitor due to their superior tolerability.

In addition to providing superior tolerability over ACE inhibitors, clinical trials have also demonstrated that the ARBs, in particular telmisartan, provide superior BP lowering to ACE inhibitors in the early morning as well as in the 24-hour, morning, daytime and night-time periods [45-50].

Hyperlipidaemia is another prevalent condition in hypertensive patients. Clinical data indicate that ARBs have no effect on lipid metabolism and are therefore safe to use in patients with hyperlipidaemia [51,52], supporting their selection in combined therapy for a broad patient population. In relation to safety, one analysis suggested that ARBs may be associated with a modest risk of lung cancer [53]; however, more complete analyses of current data have refuted this statement [54,55].

Finally, there is a wealth of data supporting the use of ARBs in diabetic patients [56], adding to the rationale for selecting this drug class for combined treatment in diabetic patients with renal impairment. ARBs and ACE inhibitors are considered equivalent in patients with type II diabetes mellitus (T2DM) with microalbuminuria. However, in patients with T2DM with proteinuria and/or renal insufficiency, ARBs are recommended because randomised controlled trials have shown that ARBs delay the progression of nephropathy in these patients [57]. Furthermore, clinical data suggest that ARBs may delay development of diabetes in at-risk patients and therefore prevent CV events in high-risk patients [56,57].

The other RAS blocker for consideration is aliskiren, a direct renin inhibitor. SPCs comprising aliskiren with a CCB or diuretic are also available. Data suggest DRIs and conventional RAS inhibitors exert similar levels of BP control [58]. However, unlike ACE inhibitors and ARBs, there is currently very limited data on the effect of aliskiren on CV and renal outcomes. The ALiskiren Trial in Type 2 diabetes Using carDio-renal Endpoints (ALTITUDE) study aimed to assess the effectiveness of alikiren in reducing CVr and renal events in patients with T2DM [59], but it was stopped early due to lack of efficacy and increased side effects, such as non-fatal stroke, renal complications, hyperkalemia and hypotension. Ongoing studies will hopefully provide these much-needed data. As there is currently little evidence to support DRI use in this patient population, we will not consider it further in this review.

### What are the preferred partners for ARB-based combinations, and why might telmisartan be a preferred ARB choice?

Most currently available ARB-based SPCs in Europe combine an ARB with either the thiazide diuretic, hydrochlorothiazide (HCTZ), or the CCB, amlodipine (Table 2) [23]. A number of clinical trials have demonstrated the superior antihypertensive efficacy of ARB/HCTZ combinations [60-63] and ARB/CCB combinations [40,64-68] compared with monotherapy.

**Table 2:** Currently authorised ARB-based two-drug SPC antihypertensive therapy in Europe in 2011 [69]

ARB	HCTZ combination	CCB combination
Telmisartan	✓	✓

Valsartan	✓	✓

Olmesartan	✓	✓

Losartan	✓	

Irbesartan	✓	

Candesartan		

Eprosartan		

Azilsartan		

Abbreviations: ARB = angiotensin II receptor blocker; CCB = calcium channel blocker; HCTZ = hydrochlorothiazide; SPC = single-pill combination

Currently, there are eight ARBs marketed for hypertension: azilsartan, candesartan, eprosartan, irbesartan, losartan, olmesartan, telmisartan and valsartan. Owing to their molecular differences, these agents demonstrate considerable variation in their pharmacokinetic and pharmacodynamic properties, which are likely to affect clinical efficacy [70]. These differences relate to lipophilicity, volume of distribution, bioavailability, biotransformation, plasma half-life, receptor affinity and residence time, as well as elimination [70,71]. The long-lasting antihypertensive effects of telmisartan compared with other ARBs are likely due to this agent having the longest plasma elimination half-life of approximately 24 hours (Table 3), as well as the highest affinity for the AT_1 _receptor [70-72]. As the most lipophilic of the ARBs, telmisartan also has the highest volume of distribution, which facilitates tissue/organ penetration (Table 3) [70-73]. Moreover, as a partial agonist of peroxisome proliferator-activated receptor-gamma, telmisartan may offer advantages in patients with insulin resistance and glucose intolerance, as well as hypertension [74,75]. These unique characteristics of telmisartan manifest in a number of clinical advantages, such as long-lasting BP control and CV protection - consequently telmisartan has been identified as a gold-standard treatment and has been recommended as a preferred ARB treatment option [76,77]. Furthermore, telmisartan has been recognised as an important therapeutic option for type 2 diabetes patients in the optimisation of CV and renal prevention [78]. These endorsements nominate telmisartan as the preferred ARB choice in combination therapy.

**Table 3:** Pharmacokinetic properties of ARBs [49,50,79]

	t_max_(h)	Bioavailability (%)	T_1/2_ (h)	Vd (L)	Interaction with food	Hepatic elimination (%)
Candesartan	3.0-5.0	42	9-13	0.13 (L/kg)	No	67

Eprosartan	2.0-6.0	13	5-7	308	No	90

Irbesartan	1.0-2.0	60-80	12-20	53-93	No	80

Losartan	1.0 (3.0-4.0)^1^	33	2 (4-6)^1^	34 (12)^1^	No	60

Olmesartan	1.4-2.8	26^2^	11.8-14.7	15-20	No	51-66^3^

Telmisartan	1	43	24	500	No	> 98^4^

Valsartan	2	23	7	17	No	83

Azilsartan	1.5-3.0	60	11	16	No	55

Abbreviations: ARB = angiotensin II receptor blocker; t_1/2 _= terminal elimination half-life; t_max _= time to maximum plasma concentration; Vd = volume of distribution

^1 ^Values in parentheses are for the active metabolite of losartan

^2 ^For olmesartan medoxomil

^3 ^Based on urinary recovery rate for intravenous olmesartan

^4 ^Faecal recovery for telmisartan

Several studies have demonstrated the superiority of telmisartan compared with other ARBs regarding 24-hour BP-lowering efficacy, particularly in the early morning period [80-86]. When a smoothness index was used to evaluate the 24-hour antihypertensive efficacy of several agents, telmisartan 80 mg had a significantly higher smoothness index than the ARBs losartan and valsartan and the ACE inhibitor, ramipril, and was comparable with amlodipine [87]. Telmisartan effectively reduces BP when used alone [32,34,88,89] or in combination with HCTZ [86,90-94] or amlodipine [37,95,96]. Telmisartan/HCTZ has demonstrated superiority over losartan/HCTZ in patients with essential hypertension in terms of 24-hour ambulatory BP, including a BP-lowering effect during the last 6 hours of the dosing interval [92,97,98]. In the Study of Micardis^® ^on Obese/Overweight Type-II diabetics with Hypertension (SMOOTH^®^), telmisartan/HCTZ demonstrated significantly greater reductions in mean ambulatory BP over the entire 24-hour dosing interval and during the last 6 hours compared with valsartan/HCTZ [93]. In two large, placebo-controlled trials, telmisartan/HCTZ also demonstrated antihypertensive superiority over valsartan/HCTZ in patients with stages 1 and 2 hypertension [86,94].

In patients with renal impairment, there are limited data on the efficacy of telmisartan/HCTZ compared with placebo, telmisartan monotherapy or other ARB-based combinations. The Diabetics Exposed to Telmisartan And enalaprIL (DETAIL^®^) study, in which more than 80% of enrolled patients had microalbuminuria, confirmed the efficacy of telmisartan in combination with a diuretic [99]. Switching patients with poorly controlled hypertension and mild-to-moderate chronic kidney disease from high-dose ARBs to telmisartan 40 mg/HCTZ 12.5 mg provided additional BP reductions and reduced urinary protein excretion, suggesting the combination is effective in this patient population [100]. Telmisartan/HCTZ has also demonstrated excellent tolerability. A retrospective safety analysis of 50 studies that evaluated telmisartan either as monotherapy or combined with HCTZ confirmed that the addition of HCTZ did not have a negative impact on the excellent tolerability profile of telmisartan, which is comparable with placebo [101]. Similar tolerability profiles have been reported for other ARB/HCTZ combinations [102-104].

Studies have also confirmed the therapeutic advantages of telmisartan and amlodipine combined therapy versus the monotherapies on reaching and maintaining BP goals in hypertensive patients [37,95,96,105]. Subgroup analysis of a trial conducted in patients with moderate-to-severe hypertension demonstrated that the telmisartan/amlodipine combination yielded reductions in mean seated trough systolic BP (SBP)/diastolic BP of up to -25.7/-19.5 mmHg in patients with mild renal impairment (estimated glomerular filtration rate [GFR] ≥ 60 ml/min/1.73 m^2^) and -26.5/-20.8 mmHg in patients with moderate-to-severe renal impairment (estimated GFR < 60 ml/min/1.73 m^2^) [106]. The BP goal of < 140/90 mmHg was achieved in up to 76.6% of patients with mild renal impairment and in up to 75.0% of those with moderate-to-severe renal impairment [106]. In a separate 8-week, randomised, double-blind trial in patients with T2DM and stages 1 or 2 hypertension (SBP > 150 mmHg), the telmisartan/amlodipine combination was superior as initial therapy compared with amlodipine 10 mg [107]. The BP goal of 140/90 mmHg was reached by 71.4% of patients treated with the telmisartan/amlodipine SPC compared with 53.8% of those treated with amlodipine 10 mg alone. For the more stringent BP goal of ≤ 130/80 mmHg, these rates were 36.4% and 17.9% for the telmisartan/amlodipine and amlodipine 10 mg groups, respectively.

**Figure 1:**
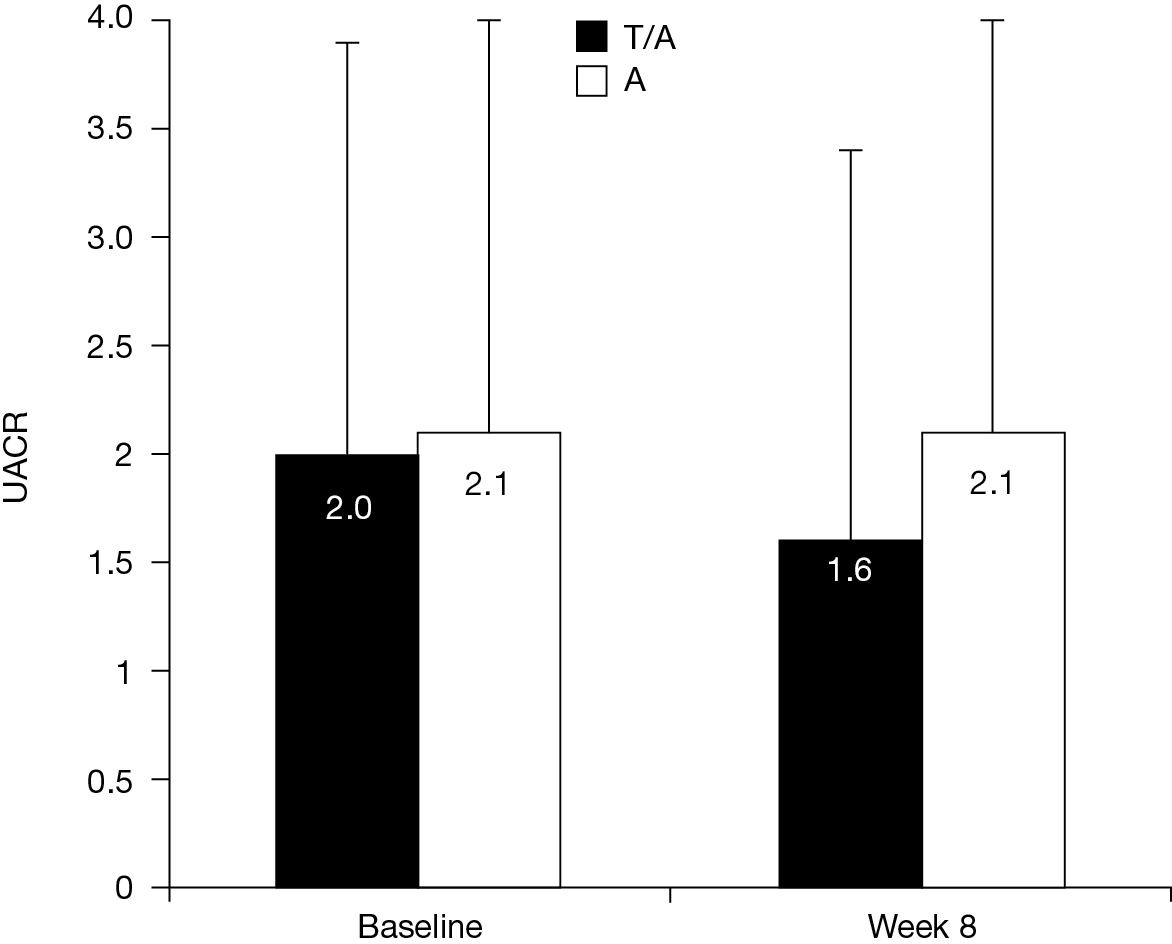
**The renal effects of amlodipine and telmisartan/amlodipine SPC**. UACR changes after 8 weeks' treatment with the telmisartan/amlodipine SPC or amlodipine monotherapy in diabetic, hypertensive patients [107]. Abbreviations: SPC = single-pill combination; T/A = telmisartan/amlodipine; UACR = urine albumin-to-creatinine ratio.

Further to the beneficial outcomes on BP, the addition of telmisartan has been shown to reduce the incidence of peripheral oedema induced by amlodipine [37]. This effect is thought to be mediated by the reduction of CCB-induced renal hyperfiltration and proteinuria - in a recent clinical study, where a 70% decrease in the urine albumin-to-creatinine ratio (UACR) was seen in those patients treated with a telmisartan and amlodipine combination compared with amlodipine monotherapy (Figure 1) [107].

### What is the preferred combination therapy for patients with renal impairment?

RAS blockers are the recommended choice of treatment for patients with renal impairment [108]. When faced with hypertensive patients with evidence of renal damage, the physician should consider the use of an ARB-based SPC, for tolerability reasons. Choices are numerous but often result in a choice between ARB/CCB and ARB/HCTZ, and it is therefore prudent to consider the evidence for these two combination types in patients with renal impairment.

In a randomised, open-label study that compared urinary albumin excretion in 207 hypertensive patients during treatment with the ARB, olmesartan, in combination with either HCTZ or the CCB, azelnidipine, ARB/HCTZ decreased UACR significantly more. This was clearly associated with greater reductions in night-time SBP, suggesting that the differential renal effects were due to differences in BP lowering [109]. In the Avoiding Cardiovascular events through COMbination therapy in Patients LIving with Systolic Hypertension (ACCOMPLISH) trial involving 11,506 patients, treatment with the ACE inhibitor, benazepril, combined with amlodipine was associated with a significant risk reduction for renal disease progression, as well as CV disease events, compared with benazepril/HCTZ in hypertensive patients at high risk for CV events. Indeed, 2.0% of patients experienced chronic kidney disease progression in the benazepril/amlodipine group compared with 3.7% in the benazepril/HCTZ group (HR, 0.52; 95% CI, 0.41-0.65; p < 0.0001) [110]. Around 18% of patients enrolled in the ACCOMPLISH trial had an estimated GFR of < 60 ml/min/1.73 m^2^, suggestive of renal disease and 6.1% were defined as having renal disease based on serum creatinine levels or the presence of macroalbuminuria [111]. The differences in the renoprotective effects of the two combinations are unlikely to be due to differences in the level of BP control because 24-hour ambulatory BP control was comparable in the two treatment arms [112,113]. The significantly greater renoprotective effects provided by the RAS blocker combined with amlodipine rather than HCTZ are more likely due to metabolic or haemodynamic properties of the specific combination [112,113].

The view that thiazide diuretics reduce GFR and have lower efficacy in the renally impaired may also impact the efficacy and renoprotective outcome of combined therapy. Consequently, loop diuretics rather than thiazide diuretics are specifically recommended in patients with ESRD/proteinuria because they more readily increase diuresis at lower GFRs [6,7].

These findings suggest that a RAS blocker, combined with a CCB rather than HCTZ, may be the combination of choice for high CV risk hypertensive patients, such as those with coronary artery disease with or without stable angina, patients with a metabolic risk profile (e.g. diabetes, obesity or metabolic syndrome) and, in particular, those with renal disease. In addition, it should be noted that some data suggests thiazide diuretics may impair glucose homeostasis and that treatment is associated with a greater incidence of diabetes compared with other antihypertensives [114,115]. Furthermore, compared with olmesartan/HCTZ, olmesartan/amlodipine treatment was associated beneficial metabolic and inflammatory effects and a lower-risk of new onset diabetes in non-diabetic patients with metabolic syndrome [116]. These data reinforce the suggestion that ARB/CCB combinations may be a preferred treatment combination, especially in patients with concomitant metabolic disorders, such as diabetes.

The renoprotective effects of ARBs and ACE inhibitors are mediated via their ability to block RAS activity [117]. This makes RAS blockers the treatment of choice in patients with diabetic kidney disease and non-diabetic kidney disease with proteinuria [108]. For the ARBs, evidence for guideline recommendations came from a number of clinical trials (predominantly in patients with chronic kidney disease) that showed ARBs to be renoprotective, independent of their BP-lowering effects (Table 4). For example, the IRbesartan in patients with type II diabetes and MicroAlbuminuria (IRMA2) study demonstrated that irbesartan, added to other antihypertensive agents, could prevent the development of diabetic nephropathy in hypertensive patients with T2DM and persistent microalbuminuria [118]. Also in patients with T2DM, the Irbesartan in Diabetic Nephropathy Trial (IDNT) demonstrated that irbesartan significantly reduced the risk of the composite primary endpoint of a doubling of serum creatinine, ESRD or death compared with placebo and amlodipine [119]. Losartan also demonstrated renoprotective effects in the Angiotensin II Antagonist Losartan (RENAAL) study [120]. In addition, the MicroAlbunuria Reduction with VALsartan (MARVAL) study showed greater reduction in urinary albumin excretion rate with valsartan than amlodipine for the same BP reduction [106,121].

**Table 4:** Results of clinical trials indicating the renoprotective nature of ARBs

Study	Patients	n	Treatment	Duration	Principle findings
AMADEO^® ^[122]	Hypertension and diabetic nephropathy	860	Telmisartan or losartan	52 weeks	Telmisartan was superior to losartan in reducing proteinuria

CALM [25]	Type 2 diabetes with hypertension and microalbuminuria	199	Candesartan, lisinopril or both	24 weeks	Candesartan was as effective as lisinopril in reducing UACR. Combined treatment was associated with a greater reduction in UACR than monotherapeutic treatment (statistically significant versus candesartan monotherapy)

DETAIL^® ^[99]	Hypertension, Type 2 diabetes and early nephropathy	250	Telmisartan or enalapril	5 years	Telmisartan was not inferior to enalapril in providing long-term renoprotection

IDNT [119]	Hypertension and diabetic nephropathy	1715	Irbesartan, amlodipine or placebo	Mean 2.6 years	Irbesartan was superior to amlodipine and placebo in preventing the primary composite end point of: a doubling of the base-line serum creatinine concentration, the development of ESRD, or death from any cause. This was independent of BP

IRMA 2 [118]	Hypertension, type 2 diabetes and microalbuminuria	590	Irbesartan or Placebo	2 years	Irbesartan was superior to placebo in preventing diabetic nephropathy

MARVAL [121]	Diabetic nephropathy with and without hypertension	332	Valsartan or amlodipine	24 weeks	Valsartan was superior to amlodipine in reducing microalbuminuria

RENAAL [120]	Diabetic nephropathy	1513	Losartan or placebo	Mean 3.4 years	Losartan was superior to placebo in preventing increases in UACR and progression to ESRD. There was no difference in mortality

ROADMAP [123]	Type 2 diabetes with normoalbuminuria	4449	Olmesartan or placebo	Median 3.2 years	Olmesartan delayed the time to onset of microalbuminuria (statistical significance lost on adjustment for blood pressure difference)

VIVALDI^® ^[124]	Hypertension and diabetic nephropathy	885	Telmisartan or valsartan*	52 weeks	Telmisartan and valsartan provided similar renoprotection

Abbreviations: AMADEO^® ^= A trial to compare telMisartan 40 mg titrated to 80 mg versus losArtan 50 mg titrated to 100 mg in hypertensive type 2 DiabEtic patients with Overt nephropathy; ARB = angiotensin II receptor blocker; BP = blood pressure; CALM = CAndesartan and Lisinopril Microalbuminuria; DETAIL^® ^= Diabetics Exposed to Telmisartan And enalaprIL study; ESRD, end-stage renal disease; IDNT = Irbesartan type II Diabetic Nephropathy Trial; IRMA2 = IRbesartan in patients with type 2 diabetes and MicroAlbuminuria; MARVAL = MicroAlbuminuria Reduction with VALsartan trial; RENAAL = Reduction of Endpoints in NIDDM with the Angiotensin II Antagonist Losartan; ROADMAP = Randomized Olmesartan And Diabetes Microalbuminuria Prevention; UACR = urine albumin:creatinine ratio; VIVALDI^® ^= A trial to inVestigate the efficacy of telmIsartan versus VALsartan in hypertensive type 2 DIabetic patients with overt nephropathy.

*Additional hypertensive treatment was allowed in VIVALDI^®^.

In hypertensive patients, telmisartan has demonstrated renoprotective effects. In the DETAIL^® ^study, telmisartan was not inferior to the ACE inhibitor, enalapril, in providing long-term renoprotection as measured by change in GFR in patients with T2DM [99]. The inVestigate the efficacy of telmIsartan versus VALsartan in hypertensive type II DIabetic patients with overt nephropathy (VIVALDI^®^) study demonstrated that telmisartan and valsartan provided similar levels of renoprotection in T2DM patients with overt nephropathy, as measured by changes in 24-hour urinary protein excretion rate, 24-hour urinary albumin excretion rate and estimated GFR [124]. In contrast, telmisartan demonstrated superior efficacy in reducing proteinuria compared with losartan, despite similar BP reductions in hypertensive T2DM patients with overt nephropathy [122].

Telmisartan has also shown efficacy in non-hypertensive patients. Based on the findings of the ONgoing Telmisartan Alone and in combination with Ramipril Global Endpoint Trial (ONTARGET^®^), which randomised 25,620 patients with vascular disease or diabetes with end-organ damage, to receive either telmisartan or the reference standard ACE inhibitor, ramipril, or a combination of the two agents [32], telmisartan is the only ARB with an indication for CV prevention independent of BP, including diabetes patients with established end organ damage such as renal disease. ONTARGET^® ^demonstrated that the two agents were equally effective in reducing the primary composite outcome of CV death, myocardial infarction, stroke or hospitalisation due to heart failure (relative risk, 1.01; 95% CI, 0.94-1.09), but that telmisartan was better tolerated than ramipril [32]. Previously, ramipril had demonstrated CV prevention properties in the Heart Outcomes Prevention Evaluation (HOPE) study [125]. Evidence from the ONTARGET^® ^and the Telmisartan Randomized AssessmeNt Study in ACE-I iNtolerant subjects with cardiovascular Disease (TRANSCEND^®^) trials also provides support for the renoprotective effects of telmisartan [126-128].

Guidelines recommend RAS blockers, such as ACE inhibitors and ARBs, as the treatment of choice for patients with renal impairment [108]. Other antihypertensives may be added if BP is not controlled. In addition, the issue of tolerability and adverse events, particularly the occurrence of ACE inhibitor-induced cough, supports the use of ARBs rather than ACE inhibitors in combination therapy in patients with renal impairment [129].

## Summary and conclusion

It is now accepted that most hypertensive patients will not reach and maintain BP goal on monotherapy. Therefore, initial combination therapy is being increasingly used and recommended by guidelines, particularly for patients with CV risk factors, such as a history of prior CV events, comorbid diabetes mellitus, microalbuminuria and evidence of organ damage, such as renal disease [7]. Guidelines also recommend the use of SPCs over free-drug combinations due to their improved adherence [7]. In patients with evidence of renal disease or in those with a greater risk of developing renal disease, such as those with diabetes and high-normal BP or overt hypertension, guidelines clearly recommend RAS blocker-based combination therapy due to superior renoprotective effects compared with other classes of antihypertensive agent [7]. Combinations containing an ARB rather than an ACE inhibitor may be preferred because ARBs are associated with superior tolerability, which may lead to improved adherence. In patients with T2DM with proteinuria and/or renal insufficiency, ARB-based treatment is recommended because these agents delay the progression of nephropathy (Table 5).

**Table 5:** Preferred antihypertensive agents based on subclinical organ damage, clinical events and comorbid conditions [6]

	ARBs	ACE inhibitors	CCBs	Diuretics	β-blockers
Uncomplicated hypertension	+	+	+	+	-

Renal dysfunction	+	+	-	-	-

ESRD/proteinuria	+	+	-	Loop diuretics	-

Metabolic syndrome	+	+	+	-	-

Diabetes mellitus	+	+	-	-	-

Isolated systolic hypertension in the elderly	-	-	+	+	-

Abbreviations: ACE = angiotensin-converting enzyme; ARBs = angiotensin II receptor blockers; CCBs = calcium channel blockers; ESRD = end-stage renal disease.

Two-drug, ARB-based SPCs are available in combination with either HCTZ or amlodipine. Telmisartan, a long-acting ARB with superior 24-hour BP-lowering efficacy compared with several other ARBs, and the only ARB with an indication for the prevention of CV disease progression, is available in two SPC formulations: telmisartan/HCTZ and telmisartan/amlodipine. Reaching a decision about which of these to use in a hypertensive patient with evidence of renal impairment is difficult in the absence of clinical trial data. However, evidence from the ACCOMPLISH trial supports the use of a RAS blocker combined with a CCB, rather than HCTZ, for high CV risk hypertensive patients, such as those with coronary artery disease with or without stable angina, patients with a metabolic risk profile and particularly for those with renal disease [110,111]. Data demonstrating beneficial metabolic and inflammatory effects with ARB/CCB combined therapy (versus ARB/HCTZ therapy), may also lead to the preferred use of RAS blocker-CCB combinations to achieve further BP reductions whilst avoiding further metabolic disturbances and protecting the kidneys from further damage [116]. However, in hypertensive patients at increased CV risk requiring an antihypertensive agent that specifically reduces blood volume, the combination of an ARB to protect the kidneys and a thiazide diuretic might be the treatment of choice.

There is a wide range of antihypertensive combinations to choose from and selecting the most appropriate treatment regimen for an individual patient with, or at risk of, renal impairment must depend on a number of considerations: careful review of the patient; the pharmacokinetic/pharmacodynamics properties of the available treatment agents; and the available clinical evidence from outcome studies.

## Abbreviations

ACE: Angiotensin-converting enzyme; ACCOMPLISH: Avoiding Cardiovascular events through Combination therapy in Patients Living with Systolic Hypertension; AMADEO^®^: A trial to compare telMisartan 40 mg titrated to 80 mg versus losArtan 50 mg titrated to 100 mg in hypertensive type 2 DiabEtic patients with Overt nephropathy; ARB: Angiotensin II receptor blocker; BP: Blood pressure; CALM: CAndesartan and Lisinopril Microalbuminuria; CCB: Calcium channel blocker; CI: Confidence interval; CV: Cardiovascular; DBP: Diastolic blood pressure; DETAIL^®^: Diabetics Exposed to Telmisartan And enalaprIL; DRI: Direct renin inhibitor; ESRD: End-stage renal disease; GFR: Glomerular filtration rate; HCTZ: Hydrochlorothiazide; HOPE: Heart outcomes prevention evaluation; HR: Hazard ratio; IDNT: Irbesartan type II Diabetic Nephropathy Trial; IHSB: International Society on Hypertension in Blacks; IRMA2: IRbesartan in patients with type 2 diabetes and MicroAlbuminuria; MARVAL: MicroAlbuminuria Reduction with VALsartan; ONTARGET^®^: ONgoing Telmisartan Alone and in combination with Ramipril Global Endpoint Trial; RAS: Renin-angiotensin system; RENAAL: Reduction of endpoints in NIDDM with the angiotensin II antagonist losartan; ROADMAP: Randomized olmesartan and diabetes microalbuminuria prevention; SBP: Systolic blood pressure; SMOOTH^®^: Study of Micardis^® ^on Obese/Overweight Type-2 diabetics with hypertension; SPC: Single-pill combination; t_1/2_: Terminal elimination half-life; T2DM: Type 2 diabetes mellitus; t_max_: Time to maximum plasma concentration; TRANSCEND^®^: Telmisartan Randomized AssessmeNt Study in ACE-I iNtolerant subjects with cardiovascular disease; UACR: Urine albumin-to-creatinine ratio; Vd: Volume of distribution; VIVALDI^®^: A trial to inVestigate the efficacy of telmIsartan versus VALsartan in hypertensive type 2 DIabetic patients with overt nephropathy.

## Competing interests

The authors declare that they have no competing interests.

## Authors' contributions

MS was solely responsible for the conception and content of this review.

## Author information

Division of Nephrology and Hypertension, Department of Internal Medicine, Faculty of Medicine, American University of Beirut, Riad El-Solh, Beirut 1107 2020, Lebanon.
